# Examination of cytotoxic and antimicrobial effect of whitening toothpastes: an *in vitro* study

**DOI:** 10.2340/aos.v83.40774

**Published:** 2024-05-27

**Authors:** Numan Aydın, Aysun Kılıç Süloğlu, Neslihan İdil, Selen Öztürk, Serpil Karaoğlanoğlu

**Affiliations:** a Department of Restorative Dental Treatment, Gulhane Faculty of Dentistry, University of Health Sciences, Ankara, Turkey; b Department of Biology, Faculty of Science, Hacettepe University, Ankara, Turkey

**Keywords:** Antimicrobial effect, cytotoxicity, human gingival fibroblast, oral health, whitening toothpaste

## Abstract

**Objective:**

Toothpastes are widely used to protect oral and teeth health. This study aims to examine the cytotoxic and antimicrobial effects of whitening toothpastes.

**Methods:**

In this study, extracts were prepared according to ISO 10993-12:2021 standard (0.2 g/mL) using whitening and conventional toothpastes. The prepared extracts were added to human gingival fibroblast cell lines (HGF-1) in different dilutions (1:1, 1:2, 1:4, 1:8, 1:16, and 1:32) and a cytotoxicity test was performed. Antimicrobial analysis of toothpastes was performed on *Streptococcus mutans, Staphylococcus aureus*, and *Candida albicans* using the hole-plate diffusion method. Cell viability and microbial analysis data were examined using two-way analysis of variance (ANOVA) and Tukey post-hoc test (*p* < 0.05).

**Results:**

Toothpastes with sodium lauryl sulfate (SLS) in their composition showed statistically more toxic effects (*p* < 0.05). The activated carbon toothpastes without SLS showed over 90% cell viability after dilution. Although the dilution rate of toothpastes containing SLS increased, cell viability remained below 70%. All toothpastes used in the study showed antimicrobial effects on *S. mutans, S. aureus*, *and C. albicans*. Toothpaste containing hydrogen peroxide and SLS produced more antibacterial effects than activated carbon, blue covarine, microparticles, and conventional toothpaste.

**Conclusions:**

SLS-containing toothpastes showed more toxicity on HGF-1 cells. Toothpaste containing hydroxyapatite did not show toxic effects on HGF-1 cells. SLS, sodium lauryl sarcosinate and hydrogen peroxide in toothpastes increase antimicrobial effects.

## Introduction

The desire to have whiter teeth by preventing or cleaning extrinsic stains on teeth increases the interest in tooth whitening products [1,2]. Although there are different tooth whitening methods, whitening toothpastes are still the first option [3].

Conventional toothpastes include sodium monofluorophosphate, silicon dioxide, hydrated silica, sodium benzoate, surfactants (SLS, sodium lauryl sarcosinate, paraben, etc.), preservatives, colorants, and buffering agents [4]. Additionally, low amounts of carbamide or hydrogen peroxide [5] and recently activated carbon have been added to whitening toothpastes [6]. Corrosives added to toothpaste to remove stains not only affect enamel surfaces but also remineralized initial caries lesions, causing unwanted abrasions on the tooth surface [7,8].

Toothpastes used in oral and teeth health have teeth whitening properties because they contain hydrogen peroxide or abrasive components [9]. Hydrated silica, calcium carbonate, dicalcium phosphate dihydrate, calcium pyrophosphate, alumina, perlite, or sodium bicarbonate in whitening toothpastes are thought to reduce the intensity and appearance of discoloration by removing pigmented biofilms and chromophores from the tooth [10].

Toothpastes containing blue covarine may leave a translucent bluish layer on the tooth surface rather than having an abrasive effect. As a result of the interaction of this layer with the light reaching the tooth surface, teeth appear brighter and whiter [11].

The charcoal in toothpastes is basically a fine powder form of activated charcoal that is oxidized by controlled reheating or chemical means. It is reported that the activated charcoal in toothpastes is effective in removing external tooth stains, as it has the capacity to adsorb color pigments [6,12].

The dental plaque formed on the tooth surface consists of a wide variety of different oral microbial strains and species [13]. *S. mutans* in dental plaque is one of the major cariogenic pathogens that metabolize fermentable carbohydrates and synthesize an extracellular polysaccharide matrix that allows organisms to adhere firmly to the tooth surface and leads to decalcification of the tooth structure [14]. *C. albicans* is mainly associated with mucosal infections (oral candidiasis) [15].

Fluorine added to toothpaste regulates antimicrobial activity, reducing demineralization and increasing remineralization [16]. Recently, hydroxyapatite, which has been added to toothpastes as an alternative to fluorine, penetrates the enamel and dentin surface affected by caries attack and forms a protective layer [17].

Toothpastes have been shown to have a whitening effect on teeth [18]. However, it is reported that the substances in toothpastes may have negative effects on oral tissues [19,20]. The aim of this study was to examine the cytotoxicity of whitening toothpastes with different contents on human gingival fibroblast cell lines (HGF-1) cell lines and antibacterial effect on *S. mutans*, *S. aureus*, *and C. albicans*. The null hypothesis was that the cytotoxic and antibacterial effects of whitening toothpastes with different active ingredients would not differ.

## Materials and methods

In this study, seven toothpastes were tested: Beverly Hills Formula Perfect White (Purity Laboratories, Ireland), Colgate Optic White Expert (Colgate-Palmolive, Poland), Colgate Optic White Charcoal (Colgate-Palmolive, Poland), Splat Blackwood (Splat, Russia), Signal White Now CC (Unilever, France), 3D White Luxe (Procte & Gamble, Germany), and Colgate Total 12 (Colgate-Palmolive, Poland). The toothpastes tested are shown in Table 1. The minimum sample size was calculated according to the results from the study of Tadin et al. [21] in 2019 about the in vivo evaluation of fluoride and SLS in toothpaste on buccal epithelial cell toxicity. A power analysis was conducted, with the effect of Cohen’s size *d* = 0.835 of the mentioned study, 80% power, and 95% confidence interval, at least 28 participants total. In this study, 36 samples were prepared for each toothpaste.

**Table 1:** The type of tooth whitening technology used in each toothpaste analyzed in this study.

Toothpaste	Composition	Tooth whitening technology
Beverly Hills Formula Perfect White	Sorbitol, Aqua, Hydrated Silica, Glycerin, Potassium Nitrate, Tetrasodium Pyrophosphate, Pentasodium Triphosphate, Aroma, Cocamidopropyl Betaine, Tricalcium Phosphate (Hydroxyapatite), PEG-32, Cellulose Gum, Sodium Saccharin, Sodium Fluoride, Charcoal powder, Limonene.Contains: Sodium fluoride 0.31% w/w (1,400 ppmF)	Activated charcoal
Colgate Optic White Expert White	Glycerin, Propylene Glycol, Calcium Pyrophosphate, PEG/PPG-116/66 Copolymer, PVP, PEG-12, Tetrasodium Pyrophosphate, Sodium Lauryl Sulfate, Silica, Aroma, Sodium Monofluorophosphate, Sodium Saccharin, Phosphoric Acid, Hydrogen Peroxide, BHT, Limonene. Sodium Fluoride (1,450 ppmF)	Hydrogen peroxide
Colgate Optic White (charcoal)	Aqua, Sorbitol, Hydrated Silica, PEG-12, Tetrapotassium Pyrophosphate, Sodium Lauryl Sulfate, Aroma, Potassium Hydroxide, Cellulose Gum, Phosphoric Acid, Cocamidopropyl Betaine, Sodium Fluoride, Sodium Saccharin, Xanthan Gum, Charcoal Powder, Mice, Limonene. Sodium Fluoride (1,450 ppmF)	Activated charcoal
Signal White Now CC	Aqua, Hydrogenated Starch Hydrolysate, Hydrated Silica, PEG-32, Zinc Citrate, Sodium Lauryl Sulfate, Aroma, Cellulose Gum, Sodium Fluoride (1,450 ppm F⁻), Sodium Saccharin, PVM/MA Copolymer, Trisodium Phosphate, Sodium Hydroxide, Glycerin, Sodium Laureth Sulfate, Lecithin, Limonene, CI 74160, CI 77891. Sodium Fluoride (1,450 ppmF)	Blue covarine pigment
Splat Blackwood	Aqua, Hydrated Silica, Hydrogenated Starch Hydrolysate, Glycerin, Maltooligosyl Glucoside, Sodium Lauroyl Sarcosinate, Cellulose Gum, Aroma, Charcoal Powder, Capryloyl/Caproyl Methly Glucamide, Lauroyl/Myristoyl Methly Glucamide, Sodium Benzoate, Stevia Rebaudiana Leaf Extract, Potassium Sorbate, Menthol, o-Cymen-5-ol, Juniperus Communis Sprout, Limonene.	Activated charcoal
Ipana 3D White Luxe	Glycerin Hydrated Silica, Sodium Hexametaphosphate, Aqua, PEG-6, Aroma, Trisodium Phosphate, Sodium Lauryl Sulfate, Carrageenan, Cocamidopropyl Betaine, Mica, Sodium Saccharin, Sodium Fluoride, PEG-20M, Xanthan Gum, CI 77891, Sucralose, Limonene, Sodium Benzoate, Sodium Hydroxide, Silica, CI 74160, Citric Acid, Sodium Citrate, BHT, Potassium Sorbate. Sodium Fluoride (1,450 ppmF)	Micro particles
Colgate Total 12	Glycerin, Hydrated Silica, Aqua, Aroma, Sodium Lauryl Sulphate, Arginine, Zinc Oxide, Cellulose Gum, Poloxamer 407, Zinc Citrate, Tetrasodium Pyrophosphate, Xanthan gum, Benzyl Alcohol, Cocamidorpropyl Betaine, Sodium Fluoride, Sodium Saccharin, Sucralose, CI 747260, CI 88891. 0.32% w/w Sodium Fluoride (1,450 ppmF)	-

### Preparation of extracts

The toothpaste samples were prepared according to ISO 10993-12:2021 [22]. After the toothpastes were placed on 24-well plates (0.2 g/mL), 5 mL of Dulbecco’s Modified Eagle Medium (DMEM) was added and incubated in the dark at 37°C for 24 hours. Then, the original solutions were centrifuged at 5,000 rpm and sterilized with 22 μm filters. The filtered 1:1 toothpaste extract was used in the cell culture in dilution series (1:2, 1:4, 1:8, 1:16, and 1:32).

#### Cell culture

Human gingival fibroblast cell lines (HGF-1, American Type Culture Collection, Manassas, VA) were routinely transplanted at 37°C and 5% CO_2_ in DMEM supplemented with 10% fetal bovine serum, penicillin and streptomycin. The cells were incubated for 24 hours with 1 × 10^4^ cells/well in 96-well plates. Spectrophotometric readings indicate the level of cellular metabolic activity. This activity represents the inhibition of succinyl dehydrogenase activity through contact between cells and toothpaste solutions. In the study, toothpaste extracts (1:1, 1:2, 1:4, 1:8, 1:16, and 1:32) were left to incubate for 2 minutes (recommended and applied average tooth brushing time) with 200 μL on the cells. It was then washed with phosphate-buffered saline (PBS) to neutralize the other effects of toothpastes on cells.

#### Cytotoxicity test

The cell viability rate of the cells was determined using MTT analysis (3-(4,5-dimethylthiazol-2-yl)-2,5-diphenyltetrazoliumbromid). Using MTT solution (Sigma-Aldrich, St. Louis, USA) of 100 μL, which was added to each well, the cells were incubated for a duration of 4 hours.The resulting formazan crystals were then dissolved by removing the culture medium before adding 100 μL of dimethyl sulfoxide solvent (Sigma-Aldrich) to each well. The plates were shaken for 10 minutes at room temperature to dissolve the crystals, and then enzyme inhibition was read using a microplate reader that utilized a spectrophotometer (Asys Hitech GmbH, Eugendorf, Austria) at 570 nm. The experiment was replicated three times. The percentage of cell viability in the experimental groups was calculated by accepting 100% of the viability in the control group.

#### Antimicrobial analysis

The antimicrobial activities of the toothpastes included in the study were determined using the hole-plate diffusion method. Overnight cultures of each microorganism sample to be tested were prepared. For this purpose, *C. albicans* ATCC 10231 was inoculated into Patoto-dextrose broth (PDB) medium, and *S. mutans* 25175 and *S. aureus* ATCC 29213 were inoculated into Brain Heart Infusion Broth (BHI) medium and incubated at 37°C for 24 hours. The turbidity of microbial suspensions for each microorganism was adjusted to 10^3^ colony forming units (CFU)/mL for ferment samples and 10^6^ (CFU)/mL for bacterial samples as a standard using sterile saline (0.9% NaCl). 100 μL of these suspensions were taken and inoculated into Müller-Hinton Agar media prepared in petri dishes by the spreading method using sterile swab. Then, a well with a diameter of 10 mm was opened for each toothpaste, and they were loaded into the wells from the samples diluted with sterile distilled water in different proportions (1:1, 1:2, 1:4, 1:8, 1:16, and 1:32). Only sterile distilled water was added to one well and used as the control. *C. albicans* cultivated petri dishes were incubated at 37°C for 48 hours, and *S. mutans* and *S. aureus* cultivated petri dishes were incubated at 37°C for 24 hours. Antimicrobial activity was evaluated by measuring the zone diameters (mm) formed after incubation. All the experiments were repeated twice.

#### Statistical analysis

The study’s data were statistically analyzed using SPSS 22.0 (SPSS Inc., Chicago, IL, USA) software. A Kolmogorov–Smirnov test was conducted to test intergroup normality, and the Levene test was conducted to test the homogeneity of variance (α = 0.05). Cell viability and microbial analysis values of toothpastes were evaluated using two-way analysis of variance (ANOVA) and Tukey post-hoc test (*p* < 0.05).

## Results

### Cytotoxicity test

**Figure 1:**
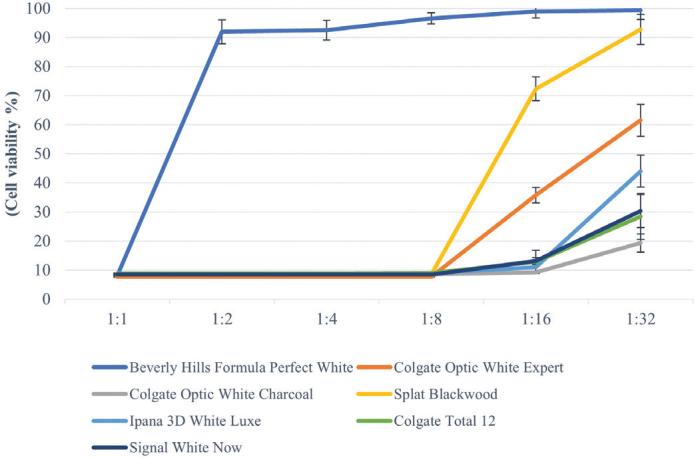
Cell viability values of toothpaste extracts at different dilutions on HGF-1 according to MTT test. The results (mean) of three independent experiments are shown as % of the control. Toothpaste without SLS showed statistically fewer toxic effects at 1:2, 1:4, 1:8, 1:16 and 1:32 dilutions (*p* < 0.05).

In this study, toothpastes without SLS showed statistically fewer toxic effects at 1:2, 1:4, 1:8, 1:16, and 1:32 dilutions (*p* < 0.05). Additionally, 1:1 extracts of all tested toothpastes showed very low cell viability (below 10%) on HGF-1 cells (Table 2). While toothpaste Beverly Hills Formula Perfect White, increased cell viability at 1:2 dilution group, other toothpastes (Colgate Optic White Expert, Colgate Optic White (charcoal), Splat Blackwood, Signal White Now, Ipana 3D White Luxe, and Colgate Total 12) showed increased cell viability at 1:16 dilution group (Figure 1).

**Table 2:** Cell viability values of toothpaste extracts at different dilutions on HGF-1 according to MTT test.

Toothpastes/ dilution rate	1:1	1:2	1:4	1:8	1:16	1:32
Beverly Hills Formula Perfect White	7.9 ± 0.1^a,A^	91.9 ± 4.1^a,B^	92.5 ± 3.4^a,B^	96.5 ± 1.9^a,B^	98.9 ± 2.3^a,B^	99.4 ± 3.2^a,B^
Colgate Optic White Expert	7.7 ± 0.1^a,A^	7.7 ± 0.1^b,A^	7.7 ± 0.1^b,A^	7.7 ± 0.1^b,A^	35.7 ± 2.7^b,B^	61.5 ± 5.5^b,C^
Colgate Optic White Charcoal	8.5 ± 0.1^a,A^	8.5 ± 0.1^b,A^	8.5 ± 0.1^b,A^	8.5 ± 0.1^b,A^	9.2 ± 0.3^c,A^	19.3 ± 3.1^c,B^
Splat Blackwood	8.5 ± 0.1^a,A^	8.5 ± 0.1^b,A^	8.5 ± 0.1^b,A^	8.5 ± 0.1^b,A^	72.4 ± 4.1^d,B^	92.7 ± 5.2^a,C^
Signal White Now	8.5 ± 0.1^a,A^	8.5 ± 0.1^b,A^	8.5 ± 0.1^b,A^	8.5 ± 0.1^b,A^	13.1 ± 1.2^c,A^	30.3 ± 5.6^c,B^
Ipana 3D White Luxe	8.9 ± 0.1^a,A^	8.9 ± 0.1^b,A^	8.9 ± 0.1^b,A^	8.9 ± 0.1^b,A^	10.9 ± 1.4^c,A^	43.9 ± 5.5^d,B^
Colgate Total 12	8.9 ± 0.1^a,A^	8.9 ± 0.1^b,A^	8.9 ± 0.1^b,A^	8.9 ± 0.1^b,A^	12.8 ± 3.9^c,A^	28.4 ± 7.9^c,B^

*The results (mean) of three independent experiments are shown as % of the control. Statistical significance value between toothpastes is shown as a–d, statistical significance value between toothpaste dilutions is shown as A–C, (*p* < 0.05).

Colgate Optic White Expert toothpaste containing hydrogen peroxide showed 40% (1:16 dilution) cell viability and 61% at 1:32 dilution group. Among the activated carbon toothpastes, 90% cell viability was observed in Beverly Hills Formula Perfect White (1:2 dilution) and Splat Blackwood (1:16 dilution), while the other carbon toothpaste Colgate Optic White (charcoal) after 1:32 dilution showed only 20% cell viability. Signal White Now containing blue covarine as a whitening agent induced 9% cell viability at 1:16 dilution and 20% at 1:32 dilution. Toothpaste with microparticles (Ipana 3D White Luxe) showed 10% cell viability at 1:16 dilution and 43% cell viability at 1:32 dilution. Conventional toothpaste (Colgate Total 12) induced 13% cell viability at 1:16 dilution and 28% cell viability at 1:32 dilution. Although the dilution rate of toothpastes containing SLS (Colgate Optic White Expert, Colgate Optic White (charcoal), Signal White Now, Ipana 3D White Luxe, and Colgate Total 12) increased (including 1:32 dilution), cell viability remained below 70%.

### Antimicrobial analysis

**Figure 2:**
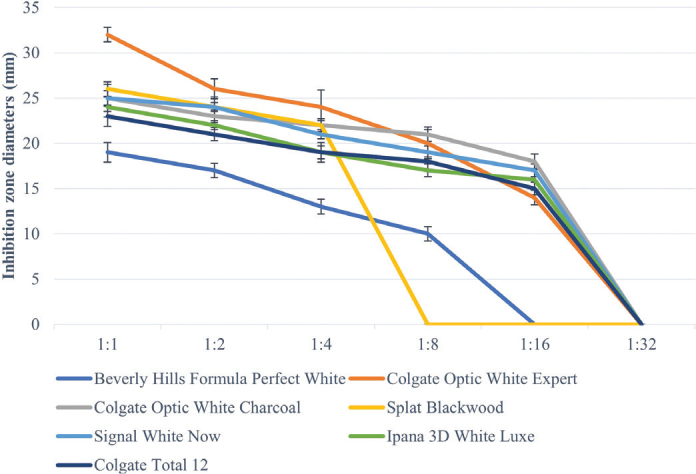
Inhibition zone diameters (mm) values of toothpaste extracts in different dilutions on *S. Mutans* according to hole-plate diffusion method.

**Figure 3:**
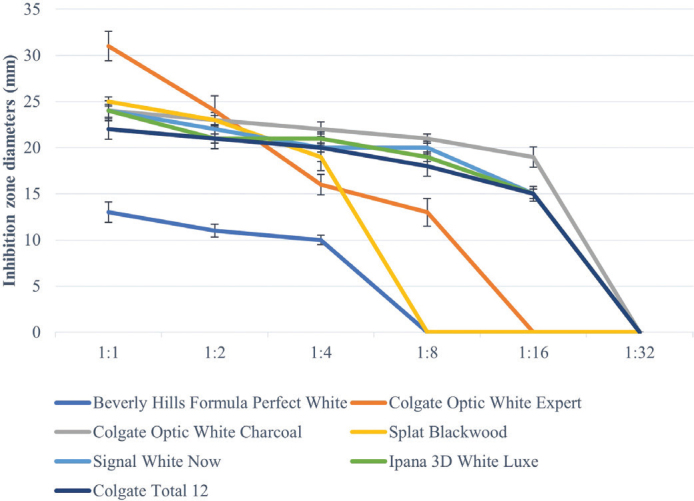
Inhibition zone diameters (mm) values of toothpaste extracts in different dilutions on *S. Aureus* according to hole-plate diffusion method.

**Figure 4:**
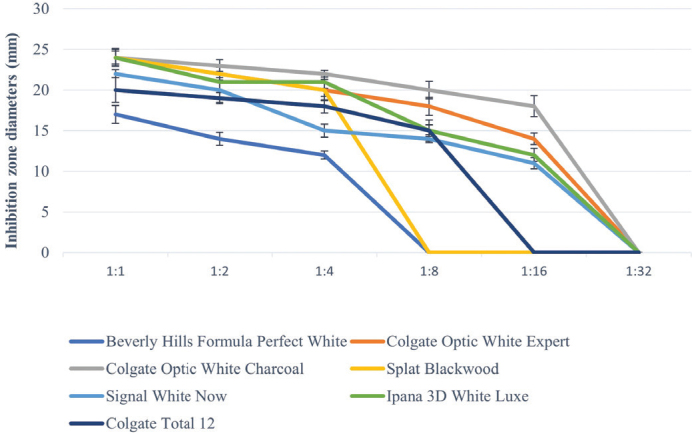
Inhibition zone diameters (mm) values of toothpaste extracts in different dilutions on *C. albicans* according to hole-plate diffusion method.

All toothpastes used in the study showed an antimicrobial effect on *S. mutans*, *S. aureus*, and *C. albicans* (Tables 3–5, Figures 2–4). Hydrogen peroxide toothpaste (Colgate Optic White Expert) showed the most antimicrobial effect on bacteria. Activated carbon and nano-hydroxyapatite containing toothpaste (Beverly Hills Formula Perfect White) produced less antimicrobial effects than other carbon-containing toothpastes (Splat Blackwood and Colgate Optic White Charcoal). Conventional toothpaste (Colgate Total 12) was found to have the same antimicrobial activity as other whitening toothpastes (except Beverly Hills Formula Perfect White). As the dilution rate of toothpastes increased (1:2, 1:4, 1:8, 1:16 and 1:32), the antibacterial effect decreased.

**Table 3:** Inhibition zone diameters (mm) values of toothpaste extracts in different dilutions on *S. Mutans* according to hole-plate diffusion method.

Toothpastes/ dilution rate	1:1	1:2	1:4	1:8	1:16	1:32
Beverly Hills Formula Perfect White	19 ± 1.1^a,A^	17 ± 0.8^a,A^	13 ± 0.8^a,B^	10 ± 0.8^a,C^	0 ± 0	0 ± 0
Colgate Optic White Expert	32 ± 0.8^b,A^	26 ± 1.1^b,B^	24 ± 1.9^b,B^	20 ± 1.5^b,C^	14 ± 0.8^a,D^	0 ± 0
Colgate Optic White Charcoal	25 ± 1.5^c,A^	23 ± 0.7^c,B^	22 ± 0.7^c,B^	21 ± 0.8^b,B^	18 ± 0.8^b,C^	0 ± 0
Splat Blackwood	26 ± 0.8^c,A^	24 ± 1.1^c,A^	22 ± 0.5^c,B^	0 ± 0	0 ± 0	0 ± 0
Signal White Now	25 ± 0.8^c,A^	24 ± 0.5^c,A^	21 ± 0.5^c,B^	19 ± 0.7^b,B^	17 ± 0.7^b,C^	0 ± 0
Ipana 3D White Luxe	24 ± 1.1^c,A^	22 ± 0.5^c,A^	19 ± 1.1^c,B^	17 ± 0.7^c,C^	16 ± 1.1^b,C^	0 ± 0
Colgate Total 12	23 ± 1.1^c,A^	21 ± 0.7^c,A^	19 ± 0.7^c,B^	18 ± 1.1^c,B^	15 ± 0.7^ab,C^	0 ± 0

*Statistical significance value between toothpastes is shown as a–c, statistical significance value between toothpaste dilutions is shown as A–D, (*p* < 0.05).

**Table 4:** Inhibition zone diameters (mm) values of toothpaste extracts in different dilutions on *S. Aureus* according to hole-plate diffusion method.

Toothpastes/ dilution rate	1:1	1:2	1:4	1:8	1:16	1:32
Beverly Hills Formula Perfect White	13 ± 1.1^a,A^	11 ± 0.7^a,B^	10 ± 0.5^a,B^	0 ± 0	0 ± 0	0 ± 0
Colgate Optic White Expert	31 ± 1.6^b,A^	24 ± 1.6^b,A^	16 ± 1.1^b,C^	13 ± 1.5^a,D^	0 ± 0	0 ± 0
Colgate Optic White Charcoal	24 ± 0.7^c,A^	23 ± 0.8^b,A^	22 ± 0.8^c,AB^	21 ± 0.5^b,B^	19 ± 1.1^a,B^	0 ± 0
Splat Blackwood	25 ± 0.5^c,A^	23 ± 0.5^b,A^	19 ± 1.5^c,B^	0 ± 0	0 ± 0	0 ± 0
Signal White Now	24 ± 1.1^c,A^	22 ± 0.5^b,A^	20 ± 0.5^c,B^	20 ± 0.7^b,B^	15 ± 0.5^b,C^	0 ± 0
Ipana 3D White Luxe	24 ± 0.7^c,A^	21 ± 0.5^b,B^	21 ± 0.7^c,B^	19 ± 0.5^b,B^	15 ± 0.5^b,C^	0 ± 0
Colgate Total 12	22 ± 1.1^c,A^	21 ± 1.1^b,A^	20 ± 1.5^c,AB^	18 ± 1.1^b,B^	15 ± 0.8^b,C^	0 ± 0

*Statistical significance value between toothpastes is shown as a–c, statistical significance value between toothpaste dilutions is shown as A–D, (*p* < 0.05).

**Table 5:** Inhibition zone diameters (mm) values of toothpaste extracts in different dilutions on *C. albicans* according to hole-plate diffusion method.

Toothpastes/ dilution rate	1:1	1:2	1:4	1:8	1:16	1:32
Beverly Hills Formula Perfect White	17 ± 1.1^a,A^	14 ± 0.8^a,B^	12 ± 0.5^a,B^	0 ± 0	0 ± 0	0 ± 0
Colgate Optic White Expert	24 ± 1.0^b,A^	22 ± 1.1^b,A^	20 ± 0.8^b,AB^	18 ± 1.1^a,B^	14 ± 0.7^a,C^	0 ± 0
Colgate Optic White Charcoal	24 ± 0.8^b,A^	23 ± 0.7^b,A^	22 ± 0.4^b,A^	20 ± 1.1^a,AB^	18 ± 1.3^b,B^	0 ± 0
Splat Blackwood	24 ± 1.1^b,A^	22 ± 0.8^b,AB^	20 ± 1.3^b,B^	0 ± 0	0 ± 0	0 ± 0
Signal White Now	22 ± 0.5^b,A^	20 ± 1.5^b,A^	15 ± 0.8^c,B^	14 ± 0.5^b,B^	11 ± 0.7^a,C^	0 ± 0
Ipana 3D White Luxe	24 ± 0.8^b,A^	21 ± 1.1^b,A^	21 ± 0.8^b,A^	15 ± 1.3^b,B^	12 ± 0.8^a,C^	0 ± 0
Colgate Total 12	20 ± 1.5^b,A^	19 ± 0.7^b,A^	18 ± 0.8^c,A^	15 ± 0.7^b,B^	0 ± 0	0 ± 0

*Statistical significance value between toothpastes is shown as a–c, statistical significance value between toothpaste dilutions is shown as A–C, (*p* < 0.05).

## Discussion

Toothpastes used for daily oral and dental care are in constant contact with the oral mucosa. Because of the potential toxicity of toothpastes, they are made available to consumers by using different names such as ‘green’, ‘natural’, and ‘organic’. As a result of the different cytotoxicity and antimicrobial effects of the toothpastes used in this study, our null hypothesis was rejected.

Although various test methods are used in studies evaluating the biocompatibility of materials, animal experiments and cell culture tests are widely used [23]. Cell culture tests are preferred because they are better standardizable and reproducible and easier to apply than animal experiments and because they are economic tests [24].

ISO 10993-12:2021 proposes several cell culture test models to evaluate the cytotoxicity of materials [22]. These are test methods of direct contact (direct method), indirect contact with a barrier (indirect method), and the method by which extracts from biomaterials are added to the cells (extract method). In this study, when the cell viability of toothpastes was evaluated by MTT test, which is an indicator of mitochondrial activity, using the extract test method on HGF-1 cells, the extracts of all toothpastes (1:1) showed toxicity. As the dilution rate increased, the toxicity decreased. These results are consistent with the results of studies revealing dose-dependent cytotoxic effects of toothpastes on HGF-1 cells [25,26].

Despite evidence that there is a low risk of toxicity and low harm when using topical fluoride appropriately [27], fear of fluoride toxicity has long been observed [28]. However, it has been stated that the total fluoride content in ppm stated on toothpastes will not always reflect the ionic fluoride ratio that occurs in brushing [29].

In studies investigating the cytotoxic effects of toothpastes *in vitro*, it was stated that all toothpastes containing SLS showed the most toxic effects [29–31]. Pecci-Lloret et al. [32] reported in their study that toothpastes containing SLS were toxic and that toothpastes containing sodium lauryl sarcosinate instead of SLS showed lower cell viability rates than those containing SLS. In their study on the cytotoxicity and genotoxicity of toothpastes on HGFs, Rode et al. [33] reported that toothpastes containing SLS or sodium lauroyl sarcosinate were significantly cytotoxic but did not show genotoxic effects.

In this study, similar to the studies in the literature [30–32], all toothpastes containing SLS showed cell viability below 70% at 1:32 dilution. Toothpaste Splat Blacwood without SLS and fluorine but containing sodium lauroyl sarcosinate showed 72% cell viability after 1:16 dilution and 90% cell viability after 1:32 dilution. Beverly Hills Formula Perfect White, the toothpaste containing fluorine and hydroxyapatite but not SLS and sodium lauroyl sarcosinate, showed cell viability above 90% after 1:2 dilution. The absence of SLS in the composition of Beverly Hills Formula Perfect White toothpaste and the added hydroxyapatite particles are considered effective in increasing cell viability.

The dominant cause of whitening effect in toothpastes is based on certain interactions between abrasives and surfactants, peroxide compounds, polyphosphates, and enzymes [34,35]. The whitening process happens by the conversion of peroxides into free radicals. In order to use this feature of hydrogen peroxide, it has been mixed in with some whitening toothpastes in low amounts [36,37]. The whitening effect of activated charcoal mixed in toothpastes serves as an effective and gradual cleaning agent for the tooth structure, because of its high capacity to adsorb and hold chromophores in the oral cavity [9,38]. Toothpastes that contain blue coverine provide the teeth look brighter and whiter by forming a translucent layer of the tooth surface [39].

In this study, the hydrogen peroxide containing toothpaste (Colgate Optic White Expert) we used showed 61% cell viability at 1:32 dilution, blue coverine containing toothpaste showed 20% cell viability at 1:32 dilution, microparticle containing toothpaste (Ipana 3D White Luxe) showed 40% cell viability, Colgate Optic White (charcoal) showed only 20% cell viability, and conventional toothpaste (Colgate Total 12) showed 30% cell viability and activated carbon containing toothpastes Beverly Hills Formula Perfect White and Splat Blackwood showed cell viability above 90%. SLS is considered to be effective on the cytotoxicity in toothpastes rather than on the whitening agents in them.

Toothpastes suppress opportunistic pathogens, such as *S. mutans*, *S. aureus*, and *C. albicans* in the regulation of oral activity in the mouth and control of dental caries and periodontal diseases [40]. The main pathogen *S. mutans* involved in the formation of dental plaque and caries was selected as the main test microorganism in this study. *C. albicans*, the most common fungal pathogen related to candidiasis, systemic infections, and even dental caries, was chosen as another pathogen for this test.

Antimicrobial activity has been evaluated using the hole-plate diffusion method, which is based on measurements of microbial inhibition sites against tested microorganisms. The diffusion event depends on the chemical and physical properties of the test substance. For example, the environment in which diffusion occurs, along with the diffusion coefficient. In addition, the bacteria colonized in dental plaque have been less susceptible to antimicrobial agents compared to planktonic bacteria [41]. As a result, this method is viable for use as a pretest to detect antimicrobial activity in products or substances.

Fluorine is widely used in toothpastes to create antibacterial effects. Fluorine affects the energy metabolism of bacteria and prevents the growth. In addition, it changes the cell membrane structure, disrupts the potassium and phosphorus balance, and provides bacterial elimination [42].

In this study, all toothpastes containing fluorine showed antibacterial effects against *S. mutans*, *S. aureus*, and C. albicans. Toothpaste containing fluorine and hydrogen peroxide showed the highest inhibition effect on *S. mutans*. In some studies, it has been reported that hydrogen peroxide has an antibacterial effect on *S. mutans* [43]. Activated carbon toothpastes produced an antibacterial effect similar to conventional toothpaste against *S. mutans*, *S. aureus*, and *C. albicans*.

Randal et al. [41] reported that the *in vitro* antimicrobial activity of toothpastes is not only dependent on the current fluoride concentration but also on the presence of other agents, such as triclosan and SLS. In our study, toothpastes without SLS showed less antimicrobial effects than those containing SLS. Splat Blackwood, one of the toothpastes, showed antibacterial effects similar to other toothpastes although it did not contain SLS and fluorine. The surfactant in this toothpaste is considered to have an antibacterial effect due to sodium lauroyl sarcosinate.

Although all toothpastes used in our study showed antimicrobial effects, toothpastes containing SLS generally showed cytotoxic effects on HGF-1 cells. However, as many features such as saliva, blood flow, gingival levels, mucus layer, and microbiota in the oral cavity, are different from *in vitro* conditions, they do not reflect *in vivo* toxic effects. It is considered that it would be beneficial to conduct clinical studies on the toxicity of whitening toothpastes in future studies.

## Conclusion

Despite the limitations of the present study, toothpastes containing SLS showed a toxic effect on HGF-1 cells, but this effect decreased as the dilution rate increased. SLS-free toothpaste did not show toxicity to HGF-1 cells. Whitening agents, such as hydrogen peroxide, activated carbon, blue covarine, and microparticles in toothpastes, did not affect the cytotoxicity of toothpastes. Whitening toothpastes have an antibacterial effect on *S. mutans*, *S. aureus*, and *C. albicans*, and adding SLS, sodium lauroyl sarcosinate, and hydrogen peroxide increases antimicrobial properties.
